# 

**Published:** 1893-09-23

**Authors:** 


**OUR NEW OFFICES,**

428, Strand, W.C., will henceforth be the Office of this Journal.

**Notices of Births, Deaths, and Marriages** are inserted in this column at a charge of 2s. 6d. for an announcement not exceeding four lines.

### NOTICE TO CORRESPONDENTS.

All MS., Letters, Books for Review, and other matters intended for the Editor should be addressed The Editor, The Lodge, Porchester Square, London, W.
The Editor cannot undertake to return rejected MS., even when accompanied by stamped directed envelope.

**Notice to Advertisers and Subscribers.**

All Advertisements, Orders for Copies of The Hospital, and Business Communications should be addressed to The Publisher, *not to the Editor*, The Hospital, 428, Strand, W.C.
The Cost of Subscription, post free, is as follows:—Per week, 2½d.; per quarter, 2s. 6d.; per half-year, 5s.; per annum, 8s. 6d. Foreign:—Per Annum, 10s. 8d.; payable, in all cases, in advance.

**"Burdett's Hospital Annual," 1893.**

Fourth year of publication. 700 pp. Boards, gilt lettering. A most complete guide to British, Colonial, Indian, and American Hospitals, Dispensaries, Nursing and Convalescent Institutions, and Asylums. Price 5s. Published by The Scientific Press (Limited), 428, Strand, W.C.

**NOTICE.—The Index for Vol. XIII. (ending March, 1893) is on sale at the Office of this Journal, price 2½d., post free.**

### SCRAPS AND GLEANINGS.

Several factories in Dartford have promised substantial support to the proposed new cottage hospital for that locality.

Mr. John Horniman has bequeathed £10,000 to the North-Eastern Hospital for Children, and £5,000 to the London Temperance Hospital.

Under a recent decision of the University of St. Andrews, lecturers of the London School of Medicine for Women are to be appointed to teach women students at the Northern University.

The Army Medical Department Report for 1891 has at length been issued. The number of men invalided as unfit for further service is considerably below the average of the past ten years.

It is being urged upon the Local Government Boards of Chiswick, Acton, and Hanwell, and on the Rural Sanitary Authority of Greenford to forthwith erect a joint infectious diseases hospital in their midst.

The capital employed in building and starting the great public kitchen for the poor of Christiana amounted to nearly £10,000. There are upwards of 3,000 meals served daily, and the cost of a dinner is 6d. a head.

The Hospital Committee of the Folkestone Town Council are contemplating considerable additions to the present arrangements at the Sanatorium. The cost of the proposed alterations is estimated at £400.

The Wandsworth Board of Works has sanctioned the erection of a fever hospital as suggested by the Metropolitan Asylums Board. A site for this object has been purchased at Grove Lane, Tooting, at a cost of £10,500.

Two local surgeon dentists have undertaken to provide a chair and attend the Llandudno Cottage Hospital professionally each morning in order that the humbler classes outside the institution may avail themselves gratuitously of their services.

The Royal Infirmary at Liverpool has just received from Mr. S. A. Yates Thompson a donation of £1,000 for the endowment of a bed, and another gift of £500 from Mr. Edward P. Thompson to be devoted to the out-patient department of the institution.

The present Infectious Diseases Hospital of the Heston and Isleworth district having proved itself imperfect in design, and insufficient in sanitary requirements, it is now proposed to erect a new hospital on more recent and approved of hygienic principles.

The statement recently issued by the Essex and Colchester Hospital Board shows a deficit of £518 on the half-yearly accounts; and it further states that whilst the subscriptions have been less than in previous years, the number of patients to the hospital have been greater.

An indignation meeting, to protest against the site selected for the proposed new fever hospital, was held recently at Alperton, "The Owners and Occupiers' Protection Society" objecting to an infectious hospital existing in such close proximity to dwelling houses.

The new fever hospital for south-east London is to be built on the Shooters Hill Road on thirty-one acres of land, purchased at £550 an acre from Lord St. Germaine. The contract for this purchase has been sealed and approved of by the Metropolitan Asylums Board.

A Bill, dealing with the amendment of laws relating to pharmacy has passed its first reading in the French Chamber of Deputies. This Bill purposes to unify the pharmaceutical diplomas, and has been introduced as a result of an agitation which has been some time on foot.

At a meeting recently held at Ampthill, Kent, a committee was appointed to consider the advisability of erecting a cottage hospital for the town. Much discouragement was given to the scheme, as it was urged that the Bedford Infirmary satisfied the requirements of the district.

In aid of the maintenance fund of the Willesden Cottage Hospital, Mrs. Passmore Edwards opened the second annual flower, fruit, and vegetable show at Willesden, on August 18th. The proceeds of this scheme last year contributed £100 towards the expenses of the institution.

A satisfactory report has just been issued by the governors of the Ingham Infirmary and South Shields Dispensary, showing on a total income of £1,900, a surplus of £45, in place of last year's deficit. A larger number of patients have received treatment than in any previous year.

We are glad to be able to congratulate the general committee of the Oxford Benefit Friendly and Trade Society's Hospital Sunday on the favourable outcome of the exertions they have put forth this year, the result of the collection exceeding the previous ones by a substantial amount.

An aquatic fête, in aid of the Denny Cottage Hospital fund, was recently held at Glencarron (by the kind permission of Mr. John Collins). The proceeds of this fête, together with the funds already in hand, forms a sufficiently large sum to warrant the commencement of the building operations.

The price of Croydon's immunity from infectious disease, so far as it can be ensured by the provision of proper isolation accommodation, will reach the startlingly high figure of £22,000. But Croydon is a rich place, and has ever been to the fore in subscribing towards the well-being of its community.

The County Councils of Oxfordshire and Shropshire have set aside a part of their finances for the purpose of training nurses for local work, including maternity and general cases. This is money well spent, and the two County Councils have established a precedent which it would be well for others to follow.

The number of in-patients at the Lewis Dispensary and Infirmary and Victoria Hospital make a total of 1,696 for the past year. In consequence of some cases of erysipelas occurring in the hospital during January last, the institution was closed to the reception of patients during a period of general disinfecting.

A special appeal is being made by the governors of Sir Patrick Dun's Hospital at Dublin, on behalf of the institution, which through diminution of its income from landed property, amounts to a yearly loss of £1,000. A number of subscriptions have been promised contingent on the full amount being made up.

The report of the governors of the North Devon Infirmary shows a deficit on the year's expenditure due to the heavy expenses of extensive repairs. The report further states that exterior financial aid has been much lessened through the general diffusion of subscriptions to the many new Cottage Hospitals in the neighbourhood.

### Medical Vacancies and Appointments.

### APPOINTMENTS.

H. Finley, M.B.Lond., M.R.C.S., L.R.C.P.Lond., appointed House Surgeon to the Cumberland Infirmary, Carlisle. F. G. Goodman, B.A., M.D.Dub., L.M.R.C.P.I., appointed Medical Officer of the Workhouse of the Glanford Brigg Union. F. W. Grant, M.D., appointed Police Surgeon to the Standing Join, Committee of the County Council of Elgin, vice Dr. Whytet resigned. W. Griffith, M.B., Ch.B., appointed House Surgeon to the London Temperance Hospital. Mr. C. Hearnden appointed Medical Officer and Public Vaccinator for the Parish of Headley of the Epsom Union. H. J. R. Jones, M.R.C.S., L.R.C.P., L.S.A., appointed Junior House Surgeon to the Poplar Hospital for Accidents. G. F. Longbotham, M.B., C.M.Edin., appointed House Surgeon to the North Riding Infirmary, Middlesbrough, vice G. V. Miller, M.B., C.M.Edin., resigned. Mr. H. H. Lovell appointed Medical Officer for the Third District of the Market Harborough Union. H. W. G. Mackenzie, M.D., M.R.C.P., M.R.C.S., appointed Assistant Physician to St. Thomas's Hospital. Mr. W. J. Proctor appointed Medical Officer and Public Vaccinator for the Great and Little Bookham Parishes of the Epsom Union, vice A. Stedman, M.R.C.S.Eng., resigned. C. G. Stein, M.B., C.M.Edin., appointed Medical Officer of Health for the Ampthill Urban Sanitary District of the Ampthill Union. J. W. Stephens, L.R.C.P.Lond., M.R.C.S.Eng., appointed Medical Officer of Health for the Cardigan Rural Sanitary District of the Cardigan Union, vice W. Davies, deceased. E. C. Stubb appointed Demonstrator of Practical Surgery at St. Thomas's Hospital. G. W. H. Tawse, M.B., C.M.Aberd., appointed House Surgeon to the Whitehaven Infirmary. G. Templeton, M.B., C.M.Edin., L.R.C.P.Lond., M.R.C.S.Eng., appointed Junior House Surgeon to the London Temperance Hospital. S. W. G. Toller, M.R.C.S., L.R.C.P., appointed Demonstrator of Practical Medicine at St. Thomas's Hospital. L. J. Weatherbe, M.B., C.M.Edin., appointed Medical Officer for the Kimberworth District and Public Vaccinator for the Rotherham and Kimberworth District of the Rotherham Union, vice B. Walker, M.R.C.S.Eng., L.S.A., resigned. G. F. Welsford, B.A., M.B.Camb., M.R.C.S.Eng., appointed Medical Officer for the Cruwys District of the Tiverton Union. R. T. Williams appointed Medical Officer of Health for the Porthcawl Urban Sanitary District of the Bridgend and Cowbridge Union. W. Bubb, M.R.C.S.Eng., L.R.C.P.Lond., appointed Third Assistant Medical Officer to the Worcester County and City Lunatic Asylum. A. B. Boyd, M.B., B.Ch.Oxon., M.R.C.S., L.R.C.P.Lond., appointed House-Physician to the Radcliffe Infirmary, Oxford. G. Brown, L.R.C.P., L.R.C.S.Edin., appointed Medical Officer of Health for the Upper Soothill Urban Sanitary District of the Dewsbury Union. A. Cameron, M.D., M.S.Glas., reappointed Medical Officer of Health for the Caistor Rural Sanitary District. W. Cholmeley, M.R.C.S., L.R.C.P., appointed House-Surgeon to the Wolverhampton and Staffordshire General Hospital. W. F. Cleaver, L.R.C.P.Lond., M.R.C.S., appointed Medical Officer of Health for the Phillack Urban Sanitary District. S. A. Coad, M.R.C.S.Eng., L.R.C.P.Lond., appointed Junior House-Surgecn to the Royal South Hants Infirmary, Southampton. F. B. H. Caudwell, L.R.C.P.Lond., M.R C.S.Eng., appointed Medical Officer for the Coggeshall District of the Braintree Union. J. Cuming, M.D.Q.U.I., reappointed Physician to the Belfast Royal Hospital. A. T. Drake, M.B.R.U.I., L.R.C.S.I., appointed Medical Officer for the Fourth Sanitary District of the Greenwich Union. Mr. G. H. Doman, appointed Medical Officer for the West Houghton District of the Bolton Union. G. T. Eccles, M.B., appointed Medical Officer of the Workhouse of the Braintree Union. J. Fagan, F.R.C.S.I., reappointed Surgeon to the Belfast Royal Hospital. F. Fowler, L.R.C.P.Lond., M.R.C.S., appointed Medical Officer for the Fourth Sanitary District of the Stroud Union. J. H. Freer, L.R.C.P.Lond., M.R.C.S., reappointed Medical Officer of Health for Rugeley. W. Galletly, M.D., L.R.C.S.Edin., appointed Parochial Medical Officer for Elgin and Public Vaccinator for the Burghal District. W. Griffith, M.B., Ch.B.Vict.Univ., appointed House-Surgeon to the London Temperance Hospital. Miss Elizabeth T. Gilchrist, L.R.C.P. and S.Edin., L.F.P.S.Glasg., appointed Resident Medical Officer in Grove Street Hospital for Women and Children, Edinburgh. R. Howden, M.B., M.S.Edin., appointed Medical Officer of Health for the Burgh of Haddington. W. G. Heasman, M.R .C.S., L.R.C.P.Lond., appointed Medical Officer for the Workho use and the Wokingham District of the Wokingham Union. A. F. Hawkins, F.R.C.S.Edin., L.R.C.P.Lond., M.R.C.S., appointed Surgeon to the Chelsea, Brompton, and Belgrave Dispensary. Mr. J. C. Hodgson, appointed Medical Officer of the Schools of the Cockerm outh Union. A. H. W. Hunt, L.R.C.P.Lond., M.R.C.S., appointed Honorary Gynæcologist to the Wolverhampton General Hospital. T. Knowles, M.B., C.M.Edin., appointed Medical Officer for the Kirkbys Malham District of the Settle Union. C. Thorne Thorne Leslie, M.R.C.S.Eng., L.R.C.P., appointed Assistant House-Surgeon to the Infirmary for Children, Myrtle Street, Liverpool. R. Stevenson, L.R.C.P., L.R.C.S.Edin., appointed Medical Officer of Health for the Rothwell Urban Sanitary District of the Hunslet Union. G. Templeton, M.B., C.M.Edin., L.R.C.P.Lond., M.R.C.S., appointed Junior House-Surgeon to the London Temperance Hospital. C. Tweedy, M.R.C.S Eng., appointed Medical Officer of Health to the Northallerton Local Board. J. H. Wallis, L.R.C.P., L.M.Edin., L.F.P.S.Glasg., appointed Medical Officer for the Igburgh Sanitary District of the Swaffham Union. M. A. Wade, L.R.C.P., L.M., L.R.C.S.I., appointed Junior Surgeon to the Police Force, Liverpool. A. W. Whitley, L.R.C.P., L.R.C.S.I., appointed Principal Medical Officer to the Chatham Dockyard. D. Watson, M.B., C.M.Glas., appointed Medical Officer for the North Welney District of the Downham Union.

### VACANCIES.

The following vacancies are announced this week, the amount of salary in each case being given after the name of the institution:

### Resident Medical Officers.

Bethlem Hospital. Two Resident Clinical Students, doubly qualified. Apartments, rations, and attendance provided. Applications, endorsed "Clinical Assistantship," to the Treasurer, at the Bethlem Hospital, by October 2nd.

County Asylum, Lancaster. Clinical Assistant. No salary. Board, &c., provided. Appointment for six months. Applications to the Medical Superintendent.

Leith Hospital. House-Physician, House-Surgeon, and Surgeon for the Out-patient Department. Appointments for six months. Salary attached to each office is at the rate of £50 per annum, with board in the hospital. Applications to the Secretary, Mr. George V. Mann, 33, Bernard Street, Leith, by September 30th.

Northumberland County Asylum, Morpeth. Clinical Clerk. Board and residence provided. Applications to Dr. McDowall, at the Asylum.

Royal Hospital for Diseases of the Chest, City Road, E.C. House-Physician. Appointment for six months. Salary at the rate of £40 per annum, with board and lodging. Applications to the Secretary by September 26th.

Seaside Convalescent Hospital, Seaford, Sussex. Resident Medical Officer. Salary £60 per annum, with board. Applications to F. Maitland, Secretary, Bank Chambers, 1, Long Acre, W.C.

Wolverhampton and Staffordshire General Hospital. House Physician, who will be required to act as Chloroformist, Pathologist, and Medical Registrar. Salary £100 per annum, with board. Testimonials to the Chairman of the Medical Committee by September 25th.

### Non-Resident Medical Officers.

Gorey Union, Killenagh and Wells Dispensary. Medical Officer. Salary £120 per annum, with £15 yearly as Medical Officer of Health, registration and vaccination fees. Applications to Mr. Benjamin Furney, Honorary Secretary, Ballycanew. Election on September 25th, when candidates must be in attendance.

National Hospital for Diseases of the Heart and Paralysis, 32, Soho Square, W. Two Clinical Assistants; doubly qualified. Applications to the Secretary by September 25th.

New Hospital for Women, 144, Euston Road, N.W. Clinical Assistant for In-patient Department. Appointment for six months. Applications to M. M. Bagster, Secretary, by September 29th.

St. Mungo's College, Glasgow. Chair of Chemistry. Applications, with thirty copies of testimonials, to the Secretary, H. Lamond, by October 2nd.

University of Aberdeen. Eleven Examiners in Medicine. The Examiners in Medicine and Surgery will receive a grant of £50 each, and the other nine a grant of £40 each. Applications to Robert Walker, Secretary, the University Court, by September 25th.

